# A comparative study of the effect of slow and rapid initiation of cardiopulmonary pump on tissue oxygenation index and ischemic complications

**DOI:** 10.1051/ject/2025050

**Published:** 2026-03-13

**Authors:** Mostafa Bagherinasab, Sahar Rezaei, Ali Reza Moradi, Ali Jabbari, Amin Noori, Zachary Archer, Nathaniel H. Darban

**Affiliations:** 1 Baqiyatallah University of Medical Sciences 19395-5487 Tehran Iran; 2 Student Research Committee, Faculty of Nursing, Tehran University of Medical Sciences 1417935840 Tehran Iran; 3 Student Research Committee, Faculty of Nursing, Baqiyatallah University of Medical Sciences 19395-5487 Tehran Iran; 4 Golestan University of Medical Science 4918936316 Gorgan Iran; 5 College of Health Sciences, Cardiovascular Science Program Glendale Arizona 85308 USA

**Keywords:** Initiation time, Cardiopulmonary bypass, Tissue oxygenation index, Ischemic complications, Near-infrared spectroscopy

## Abstract

*Introduction*: Although the use of the heart-lung machine (HLM) is routine in cardiac operating theaters, there is still a lack of evidence-based guidelines concerning the optimal speed to reach full flow during initiation to reduce critical episodes of cerebral ischemia. Therefore, we have designed a study to compare two distinct initiation times for the commencement of cardiopulmonary bypass (CPB). *Methods*: We conducted a randomized, monocentric, double-blind, prospective study to assess the impact of two different CPB initiation speeds – rapid initiation at 30 s and slow initiation at 180 s – on cerebral tissue oxygenation (TOI via NIRS), arterial oxygen pressure, hematocrit (HCT) variation, and the incidence of postoperative delirium. The target flow rate was set at 2.4 L/min/m^2^, with adjustments made according to the patient’s body surface area. *Results*: The absolute values of the tissue oxygenation index (TOI) and HCT showed no differences between the study during the first 180 s following commencement of CPB. Patients in the fast group exhibited significantly lower arterial oxygen pressure at the initiation of the (*P* < 0.05). Additionally, patients in the fast group experienced a higher incidence of delirium in the second and third days following surgery. While clinically relevant, the elevated incidence of delirium fell short of being statistically significant, with post-operative days 2 and 3 having *P*-values of 0.06 and 0.08, respectively. *Conclusion*: The results of this study indicate that, despite the absence of a significant difference in TOI between the study groups, patients in the slow group exhibited a not statistically significant trend for a lower incidence of delirium, as defined by CAMICU-7, in comparison to those in the fast group.

## Introduction

The optimal speed or time frame required to achieve complete cardiac output (CO) from the heart-lung machine (HLM) during the initiation of cardiopulmonary bypass (CPB) is not clearly defined by either the manufacturer or the scientific community [1]. Approximately three decades ago, it was reported that, in the majority of cases, complete CO or CPB flow could be achieved within 30 s [2]. The dissemination of this information has continued despite the absence of scientific validation. The initiation times vary significantly across different institutions, ranging from as short as 10 s to as long as 20 s, and in some cases, extending up to 300 s, particularly in instances such as aortic dissection [1, 3]. Theoretical considerations indicate that slower initiation times may provide neurological advantages [4]. Unlike rapid initiation, a gradual ramp-up period allows for a more gradual hemodilution, which is linked to enhanced endogenous compensation for the decreasing oxygen supply [5]. This process may consequently lead to a reduction in the incidence of neurological complications [1].

The 30- and 180-second durations were selected based on previous perfusion protocols reported in the literature and clinical feasibility [1, 4, 5]. The 30-second rapid initiation reflects conventional practice, whereas the 180-second approach was chosen to provide a distinctly gradual hemodilution process for comparative evaluation.

This research aimed to evaluate the effects of two different initiation timings – rapid and slow – on the measurements of the cerebral tissue oxygenation (TOI via NIRS) index (TOI) and the incidence of neurological complications following on-pump cardiac surgery.

## Material and methods

This randomized, monocentric, double-blind, and prospective study was carried out at the Baqiyatallah University Hospital for Cardiac Surgery over the period from October 2023 to April 2024. The research protocol received evaluation and approval from the Research Ethics Committees of Golestan University of Medical Sciences, assigned the ethics code IR.GOUMS.REC.1402.126, on July 25, 2023.

Written informed consent was obtained from all participants prior to their involvement. The inclusion criteria encompassed adult individuals aged 18 years and older who were scheduled to undergo non-emergency coronary artery bypass graft (CABG) surgery, which involved arterial cannulation of the aorta and single venous cannulation of the right atrium. Exclusion criteria included individuals with a prior diagnosis of neurological conditions, such as stroke, as well as those exhibiting any stenosis in the carotid or cerebral arteries. Furthermore, significant calcification of the aorta was another factor for exclusion. Anemia, defined as a hemoglobin level below 10 g/dL or a hematocrit (HCT) value under 30%, also disqualified certain participants. Moreover, patients requiring preoperative cardiac support systems, such as intra-aortic balloon pumps, extracorporeal membrane oxygenation, or ventricular assist devices, were considered ineligible for the study.

Demographic and medical information were collected for each patient participating in the study. Prior to the commencement of the study, a randomization process was implemented for Groups A and B, as outlined below:

(A) Cardiopulmonary bypass initiation time of 180 s is required to achieve the full target flow rate.
(B) Cardiopulmonary bypass should be initiated within 30 s to achieve the desired target flow rate.

The desired flow rate was determined based on the calculated cardiac output for each patient. This output was derived by applying a cardiac index of 2.4 L/min/m^2^, which was multiplied by the body surface area formula developed by DuBois and DuBois.

### Anesthesia and surgery protocol

All patients received anesthesia according to a standardized protocol. The perioperative management of anesthesia involved the establishment of both arterial and venous lines, monitoring via transesophageal echocardiography, and the administration of norepinephrine in conjunction with saline infusions, aimed at maintaining the mean arterial pressure within the range of 60–80 mmHg. Each patient underwent a midline sternotomy, accompanied by a standardized cannulation technique for CPB. Prior to cannulation, complete heparinization was achieved by administering 400 IU/kg of body weight in heparin, ensuring an activated clotting time (ACT) exceeding 450 s. The ACT was measured using the Medtronic HMS Plus system, employing kaolin as the activator.

### Study protocol

The configuration of our HLM was established using a LivaNova S5 (LivaNova PLC, London, United Kingdom) in conjunction with a Sorin Inspire 8F (LivaNova PLC, London, United Kingdom) oxygenator module, which features an integrated arterial filter and an open hard-shell venous reservoir. The HLM circuit was primed with 1,000 mL of Ringer’s lactate solution, which contained 10,000 IU of heparin. Prior to the commencement of extracorporeal circulation, the priming solution was warmed to a temperature of 37 °C, and the fraction of inspired oxygen was established at 0.8, with a gas flow rate of 2 L/min. In Group A, the flow rate was incrementally increased every 45 s by 25% of the total flow, ultimately achieving the desired flow rate of 100% at the 180-second mark. Similarly, in Group B, the flow rate was incrementally increased every 3.75 s by 25% of the total flow, achieving the target flow rate of 100% within 30 s. The venous clamp was adjusted to a closure of 10% to reduce the diameter of the venous conduit before the removal of the clamp on the arterial side. Subsequently, the clamp on the venous side was carefully released, ensuring that the patient remained isovolemic during the initiation of CPB, thereby preserving pulsatility throughout the measurement period.

Cerebral blood flow autoregulation is closely associated with viscosity, especially in relation to hemodilution, as well as with the partial pressure of carbon dioxide (PaCO_2_) [2]. Consequently, it is essential to alter only one variable for the purpose of comparative analysis while ensuring that PaCO_2_ levels remain constant. In this study, we maintained PaCO_2_ within the range of 30–35 mmHg. This lower range was intentionally maintained to standardize cerebral autoregulatory conditions and minimize the confounding influence of hypercapnia-induced cerebral vasodilation.

### Data collection

The demographic information collected encompassed variables such as age, sex, history of diabetes mellitus, ejection fraction, hypertension, body surface area, and preoperative creatinine levels. The evaluation of arterial blood oxygen pressure (Pao_2_), hematocrit concentrations, and fluctuations in the TOI was performed at five-minute intervals every 30 min during the first three minutes after the commencement of CPB. The B-Capta online blood gas monitoring system, developed by LivaNova, was employed to facilitate precise assessments of the PaO_2_ and temperature in the arterial line, as well as measurements of saturation, HCT, hemoglobin, and temperature in the venous line. The TOI was evaluated using NIRS (NIRO-200NX, Hamamatsu Photonics K.K., Hamamatsu City, Japan). This evaluation necessitated the placement of the appropriate electrodes on the left and right foreheads. Initial observations were conducted while the subjects were awake, prior to the administration of anesthesia, followed by subsequent measurements throughout CPB.

The perioperative and postoperative data encompassed several parameters, including aortic cross-clamp time, CPB duration, circuit priming volume, maximum CPB flow rate, duration of mechanical ventilation, and length of stay in the intensive care unit (ICU).

The Confusion Assessment Method (CAM-ICU) emerged as the predominant instrument utilized for evaluating the occurrence of delirium following surgery [6]. The CAM-ICU-7 score is quantified on a scale from 0 to 7, with a score of 7 indicating the highest severity of delirium. The scores are subsequently classified into three categories: scores ranging from 0 to 2 indicate the absence of delirium, scores from 3 to 5 reflect mild to moderate delirium, and scores between 6 and 7 denote severe delirium [7].

The Richmond Agitation-Sedation Scale (RASS) and CAM-ICU scales were evaluated twice daily, in the morning and evening, over a four-day period following the surgical procedure.

A power analysis was performed prior to study initiation. Assuming an alpha of 0.05 and a power of 80%, a minimum of 25 patients per group was required to detect a 20% difference in the primary outcome (TOI).

### Statistical analysis

Data are presented in tables as mean ± standard deviation, median, or absolute percentages. The Student’s t-test was employed for continuous parametric variables, while the Wilcoxon rank-sum test was utilized for nonparametric continuous variables. Statistical variations across the different time periods were evaluated using repeated measures analysis of variance, accompanied by Wilks’ Lambda. Results were deemed statistically significant when the associated *P*-values were below the threshold of 0.05.

## Results

### Demographic information and intraoperative and postoperative data

The demographic characteristics of the patients, along with their comorbidities, as well as intraoperative and postoperative variables, are detailed in Tables 1 and 2, respectively. No statistically significant differences were identified among the groups concerning demographic characteristics and intraoperative variables. Patients in the slow CPB initiation group exhibited a significantly reduced duration of stay in the ICU when compared to those in the fast initiation group (*P* < 0.05).

**Table 1:** Demographic data and risk factors.

Characteristic	Fast (*n* = 30)	Slow (*n* = 30)	*P*-value
Age (years)	65.16 ± 7.76	63.96 ± 7.99	0.55
Male *n* (%)	17 (56.7%)	18 (60%)	0.79
Body surface area (m^2^)	1.77 ± 0.19	1.74 ± 0.15	0.41
Ejection fraction (%)	43.00 ± 7.94	45.33 ± 7.53	0.24
Circuit priming volume	1773.33 ± 141.25	1765.00 ± 131.40	0.81
Preoperative creatinine	1.09 ± 0.23	1.10 ± 0.21	0.77
Hypertension	20 (66.7%)	19 (63.3)	0.78

Data are presented as mean ± SD, or as absolute numbers (percentage). Statistical significance was determined using Student’s t-test and the chi-square test, with a p-value threshold set at less than 0.05.

**Table 2:** Intra and postoperative data.

Characteristic	Fast (*n* = 30)	Slow (*n* = 30)	*P*-value
CPB time (minutes)	71.63 ± 21.79	70.50 ± 24.25	0.85
Aortic cross-clamp time (minutes)	51.93 ± 20.81	73.30 ± 15.72	0.46
Hemofilter (mL)	2293.33 ± 500.29	2080.00 ± 518.88	0.11
Urine output (mL)	526.66 ± 176.52	641.66 ± 355.76	0.11
Mechanical ventilation (minutes)	687.66 ± 411.61	641.00 ± 282.35	0.61
ICU stay (day)	3.90 ± 0.80	3.43 ± 0.81	0.03

Data are presented as mean ± SD, or as absolute numbers (percentage). Statistical significance was determined using Student’s t-test and the chi-square test, with a p-value threshold set at less than 0.05.

CPB = Cardiopulmonary bypass.

Key variable variations pertinent to the primary study objectives (HCT, PaO_2_, TOI) among the study groups are illustrated in Table 3. As indicated in Table 3, there were no statistically significant differences observed in HCT and TOI among the study groups during the initial 180 s. Patients in the fast group exhibited a significantly lower paO2 during the initial 180 s when compared to those in the slow group (*P* < 0.05). It is noteworthy that at baseline (T0, before anesthesia induction), the fast group already demonstrated a statistically significant lower PaO_2_ (190.7 ± 37.3 mmHg) than the slow group (233.2 ± 69.5 mmHg), with a difference of approximately 42.5 mmHg. During the initiation of CPB (T1 – T4), the PaO_2_ in the fast group remained lower than that in the slow group, but the difference (ranging from 26.6 to 32.4 mmHg) was less than the baseline difference.

**Table 3:** Key variables observed during the initial 180 s following the initiation of CPB.

Time	Fast (*n* = 30)	Slow (*n* = 30)	*P*-value
	Hematocrit		
T0	38.31 ± 4.93	37.55 ± 5.04	0.55
T1	24.12 ± 4.18	23.91 ± 3.39	0.82
T2	22.12 ± 5.37	23.81 ± 3.61	0.15
T3	23.15 ± 4.06	23.38 ± 3.34	0.81
T4	23.21 ± 3.92	23.69 ± 2.98	0.59
	Partial arterial oxygen (mmHg)		
T0	190.66 ± 37.25	233.20 ± 69.52	0.00
T1	391.70 ± 46.91	419 ± 43.35	0.02
T2	379.83 ± 47.62	412.26 ± 39.73	0.00
T3	379.56 ± 46.87	409.00 ± 35.99	0.00
T4	376.20 ± 43.99 NIRS (Left side)	402.83 ± 33.88	0.01
T0	70.56 ± 4.31	66.53 ± 5.97	0.00
T1	67.40 ± 4.44	66.43 ± 5.47	0.45
T2	67.10 ± 4.49	66.26 ± 5.20	0.50
T3	67.53 ± 4.32	66.96 ± 5.59	0.66
T4	67.83 ± 4.33 NIRS (Right side)	66.80 ± 5.31	0.41
T0	70.26 ± 4.55	68.53 ± 4.31	0.13
T1	67.00 ± 5.11	67.66 ± 4.56	0.59
T2	66.10 ± 4.76	67.63 ± 4.42	0.20
T3	67.00 ± 4.98	68.30 ± 4.22	0.28
T4	67.50 ± 4.94	68.13 ± 4.51	0.60

T0 = Before anesthesia induction, T1 = 30 s after CPB commencement, T2 = 60 s after CPB commencement, T3 = 90 s after CPB commencement, T4 = 1800 s after CPB commencement.

NIRS = Near infra-red spectrometry.

The occurrence of delirium within the study groups, assessed using the CAM-ICU, is detailed in Table 4. Patients in the slow group exhibited a not statistically significant trend for a lower incidence of delirium, particularly on the second and third days following surgery. However, these findings did not reach statistical significance (*P* = 0.06 and 0.08).

**Table 4:** Delirium occurs during the initial four days following surgery.

POD	Fast (*n* = 30)	Slow (*n* = 30)	*P*-value
First *n* (%)	15 (50%)	9 (30%)	0.11
Second *n* (%)	15 (50%)	8 (26.7%)	0.06
Third *n* (%)	11 (36.7%)	5 (16.7%)	0.08
Fourth *n* (%)	6 (20%)	3 (10%)	0.27

POD = Postoperative day.

## Discussion

Our research presents the first evidence of variations in regional brain tissue oxygenation associated with the gradual versus rapid initiation of CPB to achieve the desired target flow. While statistically significant PaO_2_ differences (~30 mmHg) were observed between groups during CPB initiation, the clinical relevance of this finding requires careful interpretation:

1. Supraphysiological oxygen levels:

Both groups maintained hyperoxic (PaO_2_ > 375 mmHg) throughout initiation – far exceeding physiological needs (normal PaO_2_: 80–100 mmHg). At these levels, oxygen content plateaus due to maximal hemoglobin saturation, diminishing the clinical impact of a 30 mmHg difference [8].

2. Potential hyperoxia risks

Emerging evidence suggests hyperoxia during CPB may increase oxidative stress and neuronal injury [9], impair cerebral autoregulation [10], and correlate with worse neurocognitive outcomes [11]. Thus, the higher PaO_2_ in the slow group (400–420 mmHg) might paradoxically confer disadvantage.

3. Physiological context:

The PaO_2_ difference represents a <1.5% change in arterial oxygen content. This minimal change is unlikely to affect oxygen delivery (DO_2_) at CPB flows >2.4 L/min/m^2^ [12].

Collectively, while the PaO_2_ difference reached statistical significance, its physiological and clinical relevance appears limited in the hyperoxic context of routine CPB management.

The results of this study indicate that, despite the absence of a significant difference in TOI and HCT between the study groups, patients classified in the slow group exhibited a clinically significant but not statistically significant lower incidence of delirium in comparison to those in the fast group.

Notwithstanding the variation in the rate of change, the absolute values concerning the impact on tissue oxygenation did not exhibit any differences at either the nadir or at 60 s after reaching 100% of the target flow rate of the HLM. This observation can be attributed to the identical dilutional volume of the priming solution utilized in both groups, as demonstrated in Table 1. Similarly, the observed disparity in NIRS values between the left and right sides, while not statistically significant, may be attributed to two potential factors. Firstly, it is plausible that the blood entering the right cerebral vessels is somewhat more diluted, as these vessels are anatomically closer to the delivery cannula of the bypass [1]. Alternatively, the jet produced by the cannula may be directed towards the left carotid artery, influencing the distribution of blood flow [1].

Severe hemodilution impacts cerebral oxygenation, necessitating increased blood pump flow rates to ensure sufficient cerebral oxygen delivery during CPB [13]. As demonstrated in Table 3, there is no statistically significant difference in HCT levels prior to the initiation of CPB. Based on the findings of this study, it can be inferred that both the fast- and slow-initiation CPB protocols are safe methods for patients, as evidenced by the aggregated numerical results pertaining to brain tissue oxygenation.

The incidence of delirium was evaluated utilizing the CAM-ICU. The CAM-ICU is a concise diagnostic tool specifically designed for the identification of delirium. It is derived from the more extensive Confusion Assessment Method (CAM), which is frequently employed in geriatric populations [14]. Notably, the CAM-ICU is distinguished by its rapid administration and its ability to be utilized without necessitating verbal communication from the patient. This feature renders it particularly suitable for application in individuals undergoing invasive mechanical ventilation and orotracheal intubation [15]. As indicated in Table 4, patients categorized in the slow group exhibited a reduced incidence of delirium in comparison to those in the fast group. The absence of a statistically significant difference may be ascribed to the limited sample size of the study. Research indicates that HCT levels and oxygen delivery significantly influence the incidence of delirium post-cardiac surgery [13, 14]. The diminished incidence of delirium noted in the slow group may be associated with lower hematocrit levels and partial pressure of oxygen (PaO_2_) at the onset of cardiopulmonary bypass (CPB). To date, there appears to be a lack of studies examining the relationship between the speed to reach full CO during CPB initiation and the subsequent development of delirium.

The duration of mechanical ventilation did not exhibit a statistically significant difference between the study groups (*P* > 0.05). Patients in the Slow group demonstrated a reduced length of stay in the ICU, which may be linked to a decreased occurrence of delirium within this study. Previous research has recognized delirium as an independent risk factor associated with extended ICU stays following cardiac surgery [13, 14].

## Conclusion

The results of this study indicate that, despite the absence of a significant difference in TOI and HCT between the study groups, patients classified in the slow group exhibited a not statistically significant trend for a lower incidence of delirium in comparison to those in the fast group.

## Limitations

This study was conducted at a single center with a relatively small sample size, which may limit the generalizability of the results. The lack of blood pressure, PaCO_2_, oxygen delivery (DO_2_), and AUC-DO_2_i data also limits the mechanistic insight. Additionally, cerebral embolization, depth of anesthesia, and postoperative medications, which may influence delirium, were not evaluated.

## Data Availability

The research data associated with this article are included within the article.
